# Exploring the Relevance between Gut Microbiota-Metabolites Profile and Chronic Kidney Disease with Distinct Pathogenic Factor

**DOI:** 10.1128/spectrum.02805-22

**Published:** 2022-12-08

**Authors:** Tso-Hsiao Chen, Chung-Yi Cheng, Chun-Kai Huang, Yi-Hsien Ho, Jung-Chun Lin

**Affiliations:** a Division of Nephrology, Department of Internal Medicine, Wan Fang Hospital, Taipei Medical University, Taipei, Taiwan; b Department of Internal Medicine, School of Medicine, College of Medicine, Taipei Medical University, Taipei, Taiwan; c Taipei Medical University-Research Center of Urology and Kidney (RCUK), School of Medicine, College of Medicine, Taipei Medical University, Taipei, Taiwan; d Department of Laboratory Medicine, Wan Fang Hospital, Taipei Medical University, Taipei, Taiwan; e School of Medical Laboratory Science and Biotechnology, College of Medical Science and Technology, Taipei Medical University, Taipei, Taiwan; f Pulmonary Research Center, Wan Fang Hospital, Taipei Medical University, Taipei, Taiwan; Huazhong University of Science and Technology; Microbiome Center, Department of Surgery, University of Chicago

**Keywords:** chronic kidney disease, diabetes mellitus, fecal metabolite, gut microbiota, hypertension

## Abstract

The intimate correlation of chronic kidney disease (CKD) with structural alteration in gut microbiota or metabolite profile has been documented in a growing body of studies. Nevertheless, a paucity of demonstrated knowledge regarding the impact and underlying mechanism of gut microbiota or metabolite on occurrence or progression of CKD is unclarified thus far. In this study, a liquid chromatography coupled-mass spectrometry and long-read sequencing were applied to identify gut metabolites and microbiome with statistically-discriminative abundance in diabetic CKD patients (*n *=* *39), hypertensive CKD patients (*n *=* *26), or CKD patients without comorbidity (*n *=* *40) compared to those of healthy participants (*n *=* *60). The association between CKD-related species and metabolite was evaluated by using zero-inflated negative binomial (ZINB) regression. The predictive utility of identified operational taxonomic units (OTUs), metabolite, or species-metabolite association toward the diagnosis of incident chronic kidney disease with distinct pathogenic factor was assessed using the random forest regression model and the receiver operating characteristic (ROC) curve. The results of statistical analyses indicated alterations in the relative abundances of 26 OTUs and 41 metabolites that were specifically relevant to each CKD-patient group. The random forest regression model with only species, metabolites, or its association differentially distinguished the hypertensive, diabetic CKD patients, or enrolled CKD patients without comorbidity from the healthy participants.

**IMPORTANCE** Gut dysbiosis-altered metabolite association exhibits specific and convincing utility to differentiate CKD associated with distinct pathogenic factor. These results present the validity of pathogenesis-associated markers across healthy participants and high-risk population toward the early screening, prevention, diagnosis, or personalized treatment of CKD.

## INTRODUCTION

The rising incidence and prevalence of chronic kidney disease (CKD) are considered essential health issues affecting around 10% of the global population (1). Hypertension, diabetes mellitus, or metabolic disorder is a widely-reported and critical risk factors toward the occurrence of CKD (2). Even though recent studies have documented the relevance of CKD progression with gender, age, or genetic signature in individual case (3, 4), persistent uncontrolled hyperglycemia, hypertension, or pro-fibrotic proteins is closely related to compromised kidney function or kidney injury, in turn resulting in end-stage renal disease (ESRD) (5). It is an urgent need for new therapeutic strategy to halt or slow the progression of CKD and reduce the increasing morbidity and mortality in this population.

During past decades, the signature or impact of gut microbiota on diverse diseases, including CKD, has been continuously disclosed (6). Recent studies revealed the constant communication of gut microbiota with distinct organs of the host, including brain, immune system, kidney and nervous system, which maintained the physiological homeostasis (7–10). Gut microbial metabolism is one source of uremic toxins that lead to renal damage, CKD deterioration and occurrence of related complications (11–13). Gastrointestinal dysfunction has been widely noted in CKD patients, subsequently resulting in the reduced diversity or alteration in the microbial community that is different from healthy people (14). Decreases in the relative abundances of probiotics with the overgrowth of pathogenic bacteria, such as Escherichia coli, *Enterococcus*, or *Fusobacterium* genera, was characterized in the gut of CKD patients (15, 16). With proliferation of these pathogenic bacteria comes the production of uremic toxins, such as Trimethylamine N-Oxide and indoxyl sulfate, which in turn aggravating CKD and intestinal barrier injury (17, 18). In contrast, supplementation of *Lactobacillus* or *Bifidobacterium* genera was demonstrated to reverse gut dysbiosis and reprogram metabolite profile, in turn restoring the intestinal barrier integrity and reducing uremic toxins in CKD animal model (19, 20). Taken together, a comprehensive understanding of the gut microbiota-metabolite axis brings an emerging insight into the therapeutic strategy throughout the progression of CKD.

In this study, the structural change of gut microbiota and metabolite profile in the patients diagnosed with hypertensive CKD (H-CKD, *n *=* *26), diabetic CKD (d-CKD, *n *=* *39), CKD patients with no comorbidity condition (NC-CKD, *n *=* *40) compared with healthy participants (HP, *n *=* *60) was identified using Oxford Nanopore Technologies (ONT) long-read sequencer and LC-QTOFMS/MS platform. Launch of the sequencing platform developed by ONT achieves single molecule real-time sequencing toward microorganism genome with sequenced reads close to 2 Mb (21). The full-length *16S rRNA* gene sequenced with the innovation confers species-level resolution toward identification of bacteria (22). Identification of the pathogenic bacteria or metabolite composition exerts potential to serve an emerging marker for the early prediction, diagnosis, or prevention of CKD occurrence or deterioration. The impact of unique species or metabolite-mediated mechanism involved in pathogenesis of CKD is worthy of further pursue in the future work.

## RESULTS

### Characteristics of the enrolled CKD patients.

A total of 165 participants were enrolled in this study, and the cohort characteristics are summarized in Table 1. Compared with the enrolled H-CKD or NC-CKD patients, the d-CKD patients showed statistically-significant elevation in fasting glucose, HbA1c, and serum creatinine (Table 1, *P < *0.05). Most H-CKD or NC-CKD patients were classified as stage 3 (Table 1, 67% and 73%), which was not noted among the d-CKD patients. There was no significant difference in the statistical results regarding estimated glomerular filtration rate (eGFR), age, or sex among all recruited CKD patients (Table 1).

**TABLE 1:** Characteristics of healthy participants and enrolled diabetic, hypertensive CKD patients, or CKD patients without comorbidity

Group	Healthy group (*n* = 60)	NC-CKD (*n* = 40)	Hypertensive CKD (*n* = 26)	Diabetic CKD (*n* = 39)	*P*
CKD stage No. (%)	-^*a*^	Stage 1 & 2: 5 (12.5%) Stage 3: 27 (67.5%) Stage 4 & 5: 8 (20%)	Stage 1 & 2: 4 (15.38%) Stage 3: 19 (73.07%) Stage 4 & 5: 13 (11.54%)	Stage 1 & 2: 10 (25.64%) Stage 3: 17 (43.6%) Stage 4 & 5: 12 (30.76%)	
Age (Median(IQR))	66 (41–87)	69 (33–90)	71 (40–88)	71 (43–90)	
Sex (*n*,%)					
Female	32 (53.33%)	21 (52.5%)	15 (57.69%)	15 (38.46%)	>0.05
Male	28 (46.67%)	19 (47.5%)	11 (42.31%)	24 (61.54%)	>0.05
Fasting Blood Glucose (mg/dL) (Median(IQR))	89 (61–100)	98 (77–157)	99 (85–166)	135 (84–425) (*P *<* *0.005)	>0.05
HbA1c (%) (Median(IQR))	5.1 (4.2–6.0)	5.7 (4.1–6.4)	5.9 (5.1–9.4)	7.05 (5.6–9.4) (*P *<* *0.005)	>0.05
Serum Creatinine (mg/dL) (Median(IQR))	0.72 (0.5–1.15)	1.4 (1.11–13.15)	1.505 (0.97–3.85)	1.615 (0.73–8.75) (*P *<* *0.05)	>0.05
eGFR (mL/min/1.73m2) (Median(IQR))	92.4 (63.9–134.2)	40 (4–83)	46 (16–69)	40 (7–84)	>0.05

a -, not applicable.

### Statistical analysis of ONT sequencing results.

In this study, the genomic DNA extracted from the fecal sample of enrolled participants was subjected to the characterization of gut microbial communities by using the long-read sequencing platform (MinION, ONT, Oxford, UK). The CLC Genomics Workbench software (v.22.0.2; Aarhus, Denmark) was applied to evaluate the average number of sequenced reads and qualified reads per sample. No statistical difference regarding the sequencing efficiency was characterized among each group (Table 2, *P > *0.05), whereas more OTUs were identified within the microbial communities in enrolled NC-CKD or d-CKD patients compared to those of H-CKD patients or healthy participants (Table 2, *P < *0.01).

**TABLE 2:** Statistical summary of sequencing throughput and identified result in each enrolled group

Group	Healthy group (*n* = 60)	NC-CKD (*n* = 40)	Hypertensive CKD (*n* = 26)	Diabetic CKD (*n* = 39)	*P*
No. of Raw reads per sample	38,786 (±2,079)	49,532 (±3,709)	48,603 (±4,719)	43,997 (±4,217)	>0.05
No. of qualified reads per sample	32,527 (±2,226)	45,289 (±3,114)	45,321 (±3,134)	40,328 (±3,050)	>0.05
Reads in identified taxa	25,329 (±1,845)	35,677 (±2,851)	37,951 (±2,977)	32,375 (±2,505)	>0.05
Correctly classified (% (SD))	77.87 (±3.64)	78.78 (±6.07)	83.74 (±6.52)	80.28 (±5.69)	>0.05
No. of identified taxa per sample	1,219	1,965(*P < *0.01)	1,021 (*P > *0.05)	1,865 (*P < *0.01)	

**FIG 1:**
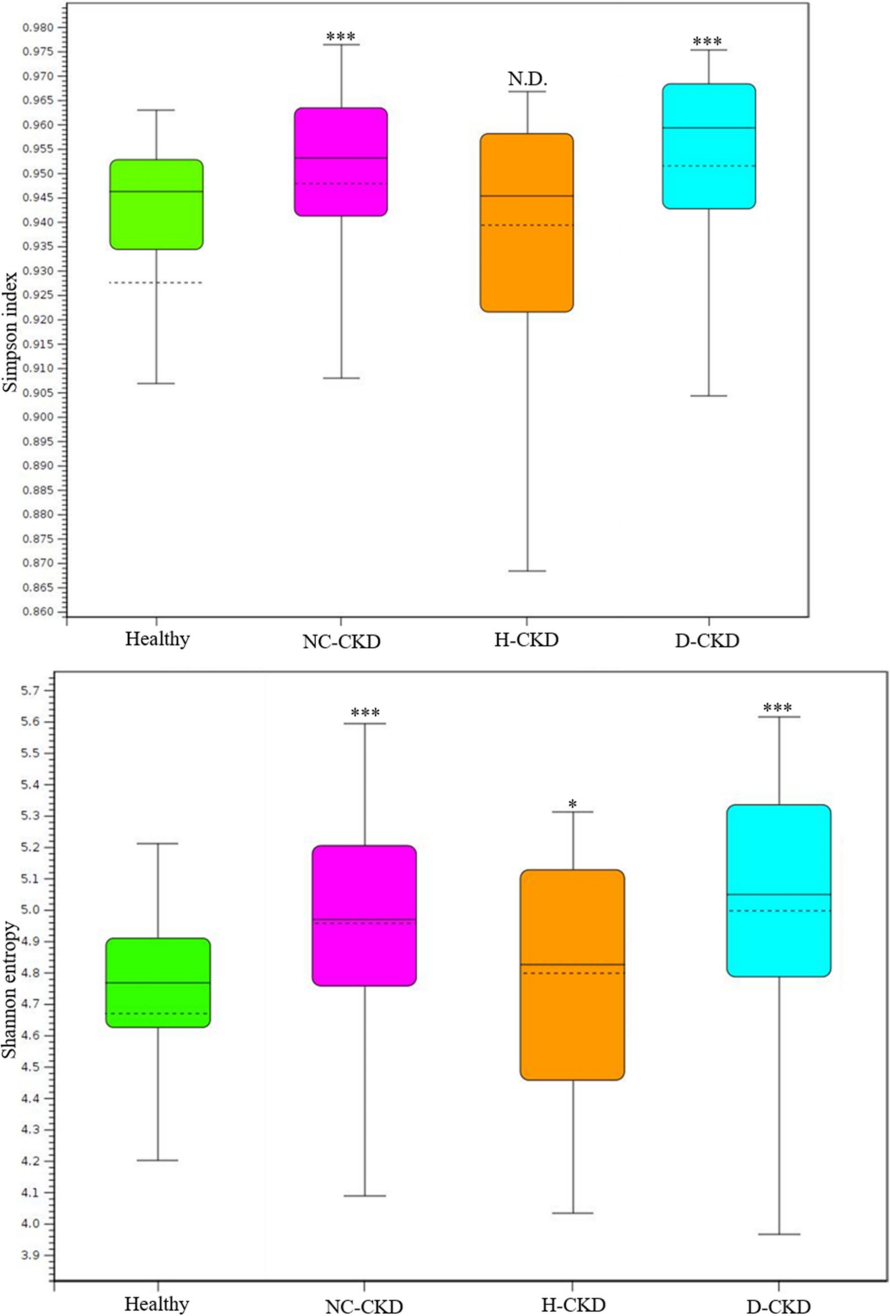
Complexity of taxonomic composition among the enrolled groups with long-read sequencing results. The α-diversity in all groups is synchronously evaluated using Simpson index (top) and Shannon entropy (bottom).

**FIG 2:**
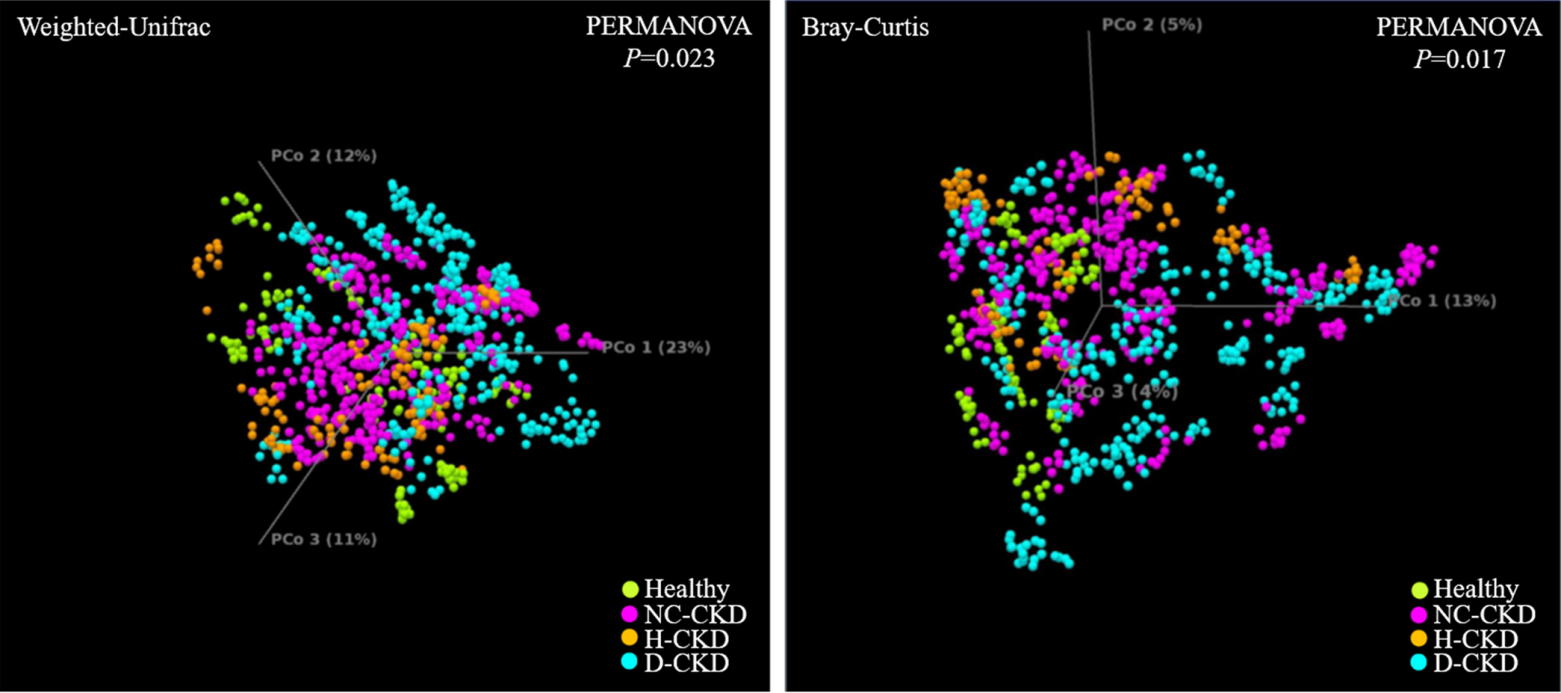
Dissimilarity in taxonomic composition among the enrolled groups is evaluated by using Weighted Unifrac principal-component analysis (PCoA) (left) and Bray-Curtis dissimilarity analysis (right).

The analytic results of Simpson index (Fig. 1, left) or Shannon entropy (Fig. 1, right) indicated difference in the species diversity (α-diversity) between the gut microbial communities in healthy participants and NC-CKD patients (*P < *0.005) or d-CKD patients (*P < *0.005), but not H-CKD patients (*P < *0.05, Shannon entropy; *P > *0.05, Simpson index). Principal coordinates analysis (PCoA) with Weighted Unifract distance (Fig. 2, left) or Bray-Curtis indices (Fig. 2, right) was applied to visualize the dissimilarity of gut microbial communities among all groups. A Weighted Unifrac or Bray-Curtis distance between each sample was applied to indicate the discrimination in the gut microbial communities among healthy participants (Fig. 2, green dot) and H-CKD patients (Fig. 2, brown dot), d-CKD patients (Fig. 2, blue dot), or NC-CKD patients (Fig. 2, pink dot). The isolated clusters within microbial communities of each CKD-patient group were shown in PCoA space (Fig. 2). The beta diversity of the gut microbial communities differed between each group of the CKD patients compared with another or healthy participants (Fig. 2, PERMANOVA *P* value = 0.023 or 0.017).

### Classification of gut dysbiosis of the enrolled CKD patients.

**FIG 3:**
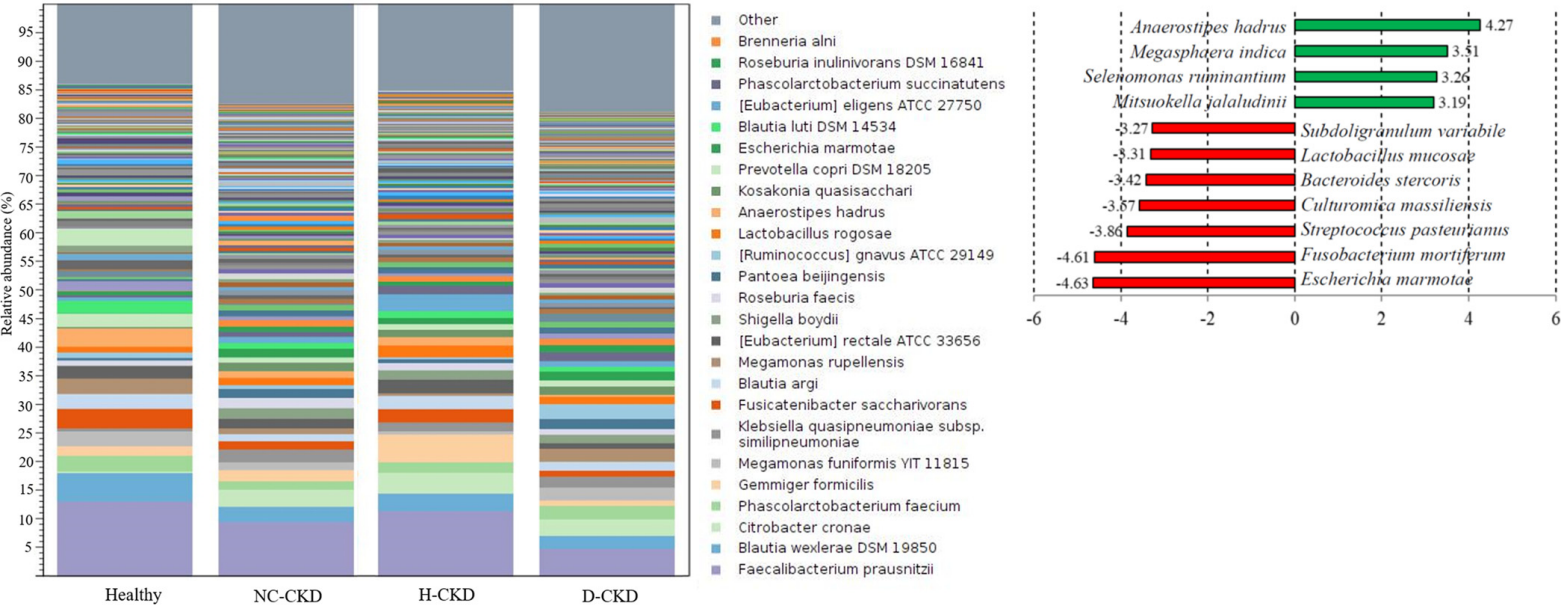
Classification of operational taxonomy unit (OTU) with long-read sequencing in healthy participants and enrolled CKD patients. The relative levels of top 25 OTUs to species level based on the average reads among all recruited group is presented in stacked bar chart (left). Linear discriminant analysis (LDA) scores indicate discriminative abundances of OTUs in healthy participants (green bar) and CKD patients (red bar) (right).

**FIG 4:**
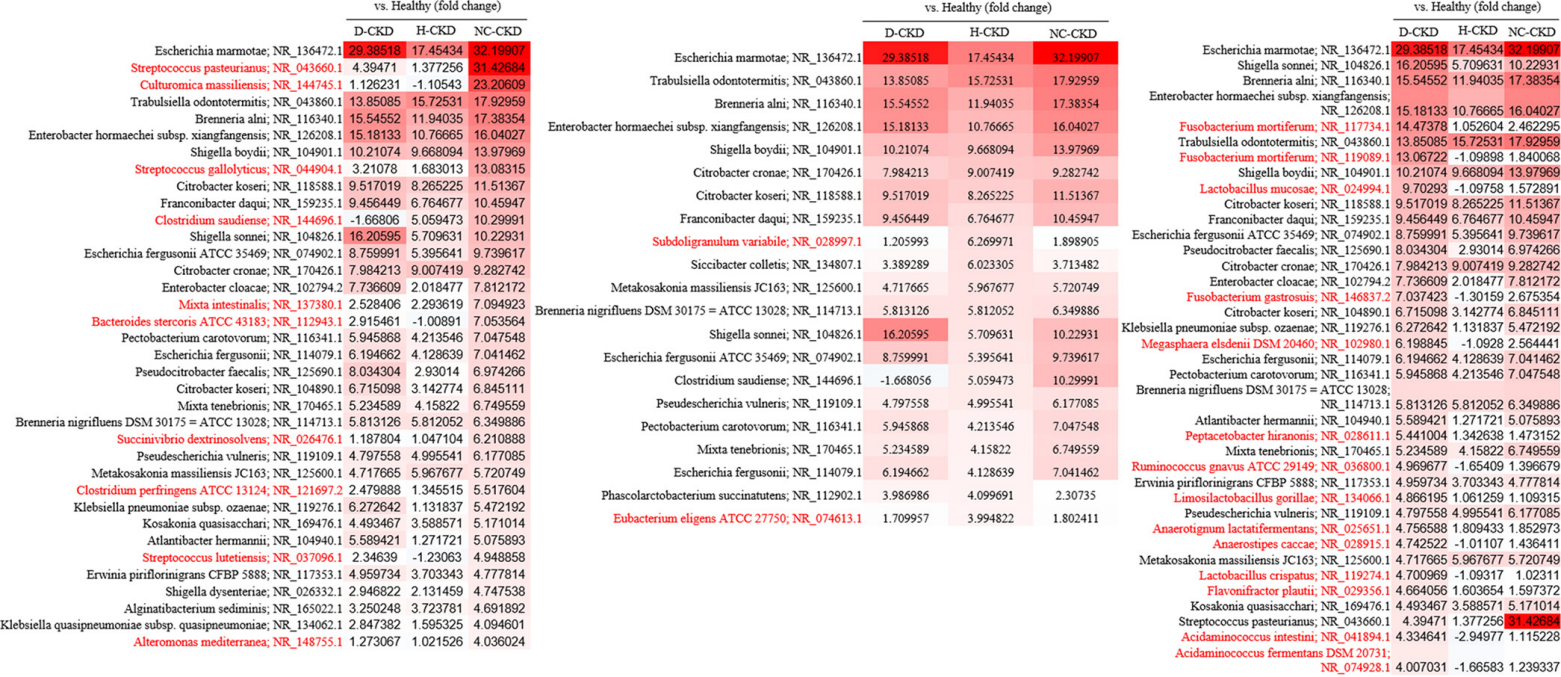
Comorbidity-associated gut microbial communities across all enrolled CKD groups. Relevance between the discriminative abundance of classified OTUs at the species level and recruited CKD patients with distinct comorbidity is shown in a heat map chart (red character).

A total of over 1,000 OTUs at the species level were classified with the usage of ONT sequencer and CLC Genomics Workbench software in this study. The top 25-ranked OTUs were shown based on the average reads that classified among all recruited group (Fig. 3, bar chart). Linear discriminant analysis (LDA) effect-size (LEfSe) analyses were conducted to discriminate the differential abundances of identified OTUs between healthy participants and CKD patients (23). The results of LDA score indicated statistically high levels of Escherichia marmotae, Fusobacterium mortiferum, Streptococcus pasteurianus, Bacteroides stercoris, Lactobacillus mucosae, *Culturomica massiliensis*, and Subdoligranulum variabile in the microbial communities of CKD patients (Fig. 3, right, red bar) compared with the healthy group (LDA score (log 10) < -3). In contrast, Mitsuokella jalaludinii, Megasphaera indica, Selenomonas ruminantium, and Anaerostipes hadrus were relatively more abundant in the gut microbiota of healthy group (Fig. 3, right, green bar; LDA score (log 10) > 3) compared to those of enrolled CKD patients. Among the CKD-related candidates, the higher abundances of Streptococcus pasteurianus, Bacteroides stercoris, and *Culturomica massiliensis* were specifically identified in the gut microbial communities of NC-CKD patients than those of other CKD patients (Fig. 4, left, red character), whereas the relatively high abundance of Escherichia marmotae was identified throughout the all CKD groups. The relatively abundant Fusobacterium mortiferum and Lactobacillus mucosae were specifically identified in the gut microbial communities of d-CKD patients (Fig. 4, right, red character). These results suggested the relevance between the structural changes of gut microbiota and the pathogenesis of CKD with discriminative disease condition.

### Discriminative metabolomic signatures of each CKD-patient group.

**FIG 5:**
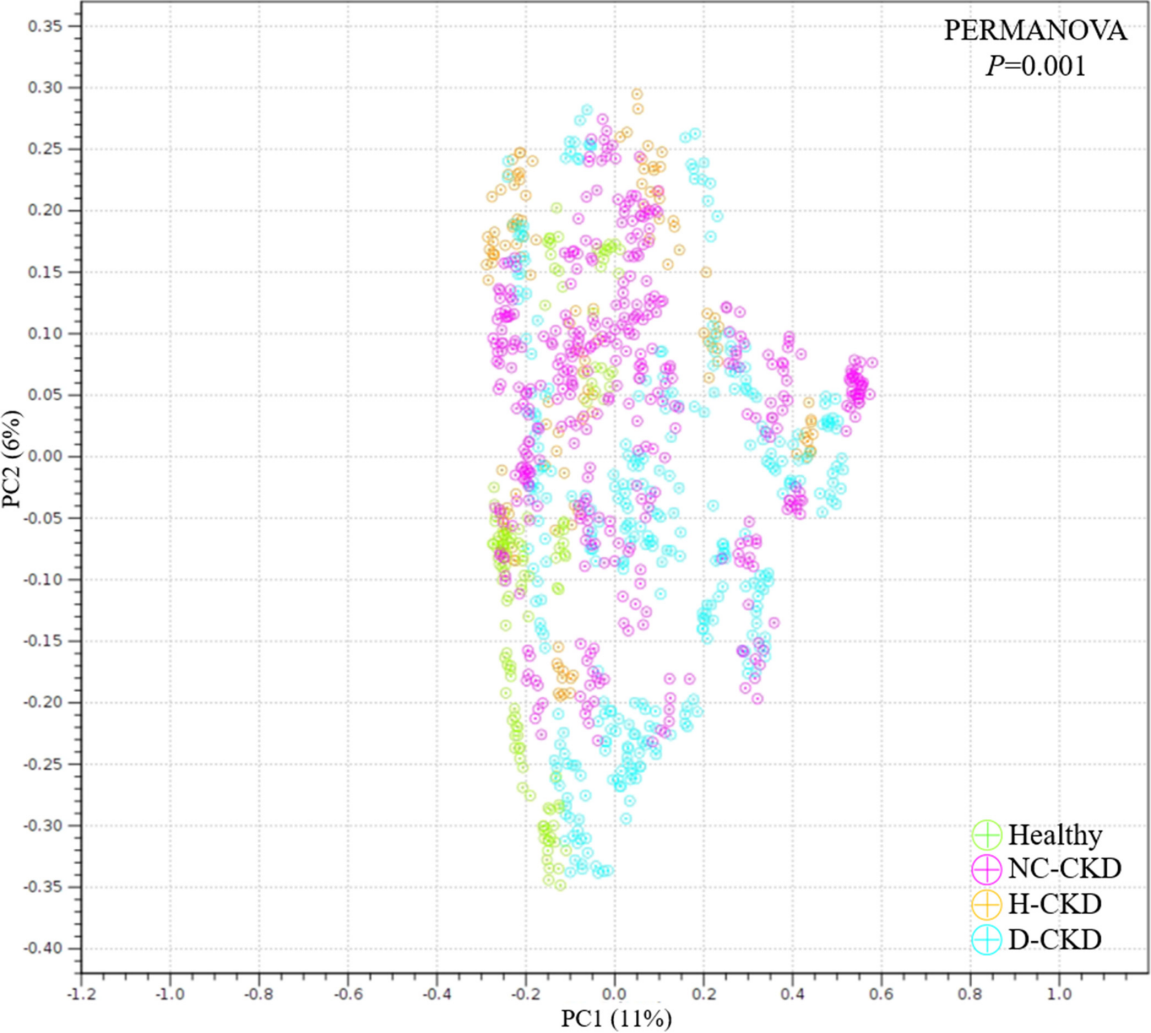
Principal-component analysis (PCA) is subjected to illustrate the dissimilarity of gut metabolomic profiling between healthy participants and enrolled diabetic CKD (d-CKD), hypertensive CKD (H-CKD), or CKD patients with no comorbidity (NC-CKD).

**FIG 6:**
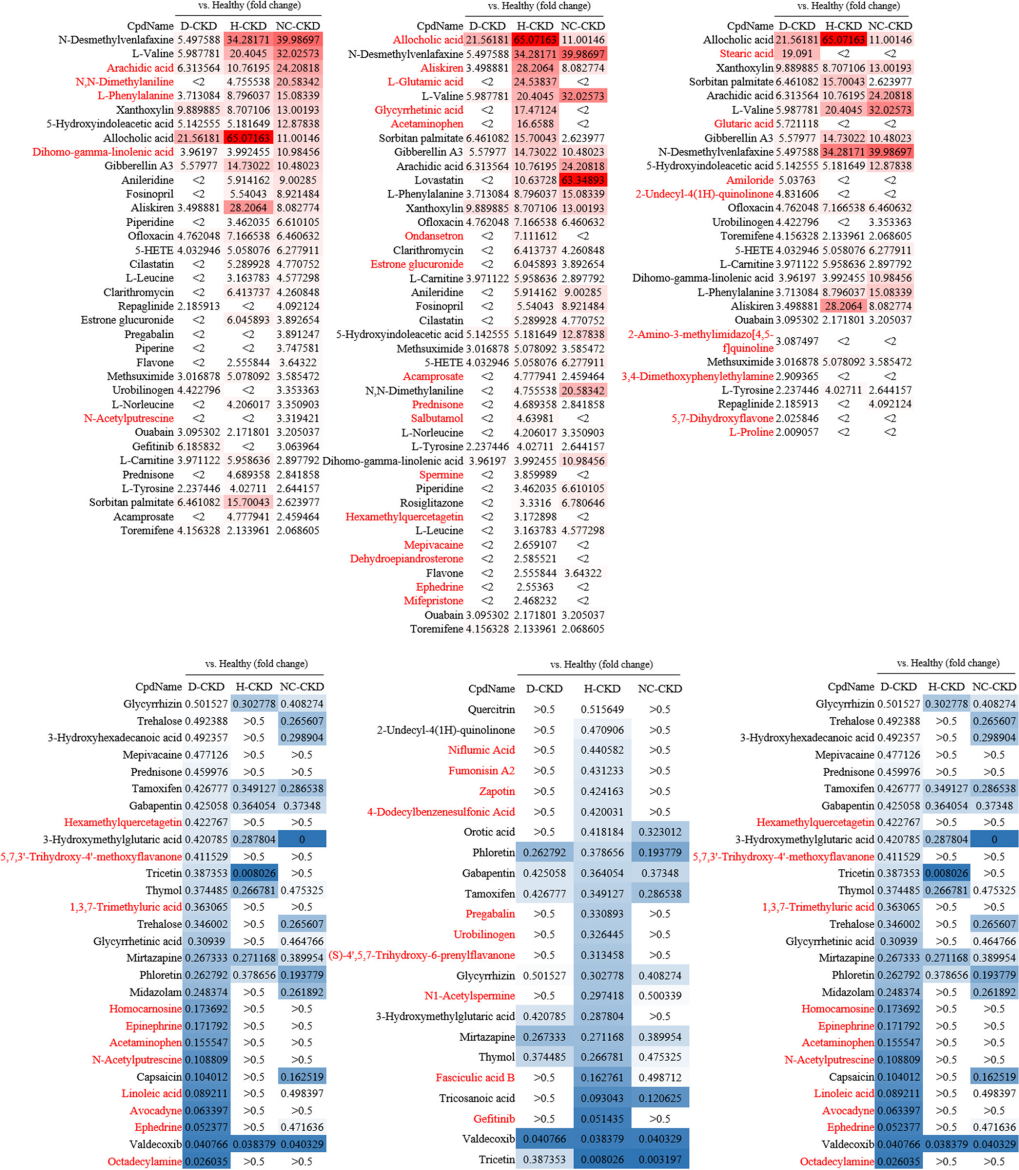
Z-score heatmap is constructed with the statistically discriminative abundances of gut metabolites identified among enrolled diabetic CKD (d-CKD), hypertensive CKD (H-CKD), or CKD patients with no comorbidity (NC-CKD). Relevance between the discriminative increases (upper panel) or decreases (lower panel) in the identified metabolites and recruited CKD patients is shown in a heat map chart (red character). Significance of listed metabolites was evaluated using the value of variable importance in projection value (VIP) and alteration in relative abundance from pairwise PLD-DA analysis and Wilcoxon rank-sum test, with VIP > 1.5, alteration in relative abundance (-2> fold change >2), and *P* value < 0.05 as the cut-off for significance.

In this study, a total of 187 gut metabolites were identified in the fecal samples of enrolled participants by using the UPLC-MS/MS platform and corresponding analytic pipeline. The principal-component analysis (PCA) was conducted to evaluate the divergence of gut metabolite profiles among all groups. As shown in Fig. 5, the isolated clusters within metabolite profiles of each CKD group were identified in PCA space (Fig. 5, PERMANOVA, *P = *0.001). The gut metabolites with discriminative abundances among healthy participants and each CKD group were characterized with the following criteria, including a significant alteration in relative abundance (-2> fold change >2), a variable importance in projection value (VIP) > 1.5, and a statistical *P* value < 0.05 (Table 3). A heat map was shown to illustrate the increased abundances of 29 metabolites (Fig. 6, upper, red character) and decreased levels of 32 metabolites in each CKD group (Fig. 6, lower, blue character). These results suggested the potential application of identified metabolite for serving the specific marker toward the occurrence of CKD with distinct pathogenic factor.

**TABLE 3:** Statistics of gut metabolites with discriminative abundance in feces samples of CKD patients compared with healthy group

Metabolite	KEGG	HMDB	Microbe	Hsa	CKD	VIP	*P* value	Fold change (CKD vs. healthy)	RSD (%)
Arachidic acid	C06425	HMDB0002212	Yes	Yes	NC	3.04	0.017	24.20818	5.61
N,N-Dimethylaniline	C02846	HMDB0001020	NA	NA	NC	2.95	0.013	20.58342	8.57
L-Phenylalanine	C00079	HMDB0000159	Yes	Yes	NC	3.12	0.008	15.08339	9.04
Dihomo-gamma-linolenic acid	C03242	HMDB0002925	Yes	Yes	NC	2.54	0.023	10.98456	11.23
N-Acetylputrescine	C02714	HMDB0002064	Yes	Yes	NC	2.21	0.027	3.319421	15.48
Hexamethylquercetagetin	NA	HMDB0029308	NA	NA	NC	1.97	0.035	0.422767	21.51
5,7,3′-Trihydroxy-4′-methoxyflavanone	NA	HMDB0030746	NA	NA	NC	2.04	0.026	0.411529	17.69
1,3,7-Trimethyluric acid	C16361	HMDB0002123	Yes	Yes	NC	1.99	0.018	0.363065	13.44
Homocarnosine	C00884	HMDB0000745	Yes	Yes	NC	2.06	0.025	0.173692	11.27
Epinephrine	C00788	HMDB0000068	Yes	Yes	NC	2.87	0.018	0.171792	10.05
Acetaminophen	C06804	HMDB0001859	NA	Yes	NC	3.12	0.017	0.155547	11.34
N-Acetylputrescine	C02714	HMDB0002064	Yes	Yes	NC	3.55	0.024	0.108809	12.65
Linoleic acid	C01595	HMDB0000673	Yes	Yes	NC	2.57	0.015	0.089211	10.91
Avocadyne	NA	HMDB0035473	NA	NA	NC	3.05	0.009	0.063397	8.77
Ephedrine	C01575	HMDB0015451	Yes	Yes	NC	3.11	0.011	0.052377	6.45
Octadecylamine	NA	HMDB0029586	NA	NA	NC	2.85	0.018	0.026035	7.23
Allocholic acid	C17737	HMDB0000505	Yes	NA	H-CKD	3.581	0.0094	65.07163	3.41
Aliskiren	NA	HMDB0015387	NA	NA	H-CKD	3.445	0.012	28.2064	7.25
L-Glutamic acid	C00025	HMDB0000148	Yes	Yes	H-CKD	3.17	0.017	24.53837	8.51
Glycyrrhetinic acid	C02283	HMDB0011628	NA	NA	H-CKD	3.04	0.021	17.47124	9.63
Acetaminophen	C06804	HMDB0001859	NA	Yes	H-CKD	2.85	0.014	16.6588	7.22
Ondansetron	C07325	HMDB0005035	NA	NA	H-CKD	2.71	0.023	7.111612	10.34
Estrone glucuronide	C11133	HMDB0004483	NA	Yes	H-CKD	2.34	0.027	6.045893	15.41
Acamprosate	NA	HMDB0014797	NA	NA	H-CKD	2.25	0.031	4.777941	17.22
Prednisone	C07370	HMDB0014773	NA	NA	H-CKD	2.12	0.022	4.689358	13.41
Salbutamol	C11770	HMDB0001937	NA	NA	H-CKD	1.97	0.027	4.63981	16.22
Spermine	C00750	HMDB0001256	Yes	Yes	H-CKD	2.05	0.034	3.859989	23.41
Hexamethylquercetagetin	NA	HMDB0029308	NA	NA	H-CKD	1.84	0.029	3.172898	27.45
Mepivacaine	C07528	C07528	NA	NA	H-CKD	1.88	0.033	2.659107	17.22
Niflumic Acid	C13698	HMDB0015573	NA	NA	H-CKD	1.84	0.042	0.440582	21.34
Fumonisin A2	NA	HMDB0034699	NA	NA	H-CKD	1.92	0.037	0.431233	19.55
Zapotin	NA	HMDB0029461	NA	NA	H-CKD	1.77	0.025	0.424163	17.21
Pregabalin	NA	HMDB0014375	NA	NA	H-CKD	2.01	0.023	0.330893	20.33
Urobilinogen	C05791	HMDB0004158	Yes	Yes	H-CKD	1.95	0.031	0.326445	21.42
(S)-4′,5,7-Trihydroxy-6-prenylflavanone	C09832	HMDB0037247	NA	NA	H-CKD	1.99	0.024	0.313458	18.57
N1-Acetylspermine	C02567	HMDB0001186	NA	NA	H-CKD	2.12	0.019	0.297418	17.24
Fasciculic acid B	NA	HMDB0036438	NA	NA	H-CKD	2.42	0.021	0.162761	15.41
Gefitinib	NA	HMDB0014462	NA	NA	H-CKD	2.28	0.013	0.051435	13.07
Stearic acid	C01530	HMDB0000827	Yes	Yes	D-CKD	3.14	0.012	19.091	11.34
Glutaric acid	C00489	HMDB0000661	Yes	Yes	D-CKD	2.94	0.017	5.721118	13.55
Amiloride	C06821	HMDB0014732	NA	NA	D-CKD	2.85	0.02	5.03763	9.81
2-Undecyl-4(1H)-quinolinone	NA	HMDB0032996	NA	NA	D-CKD	2.77	0.021	4.831606	14.52
2-Amino-3-methylimidazo[4,5-f]quinoline	C19180	HMDB0029706	NA	Yes	D-CKD	2.51	0.019	3.087497	13.77
3,4-Dimethoxyphenylethylamine	NA	HMDB0041806	NA	NA	D-CKD	2.24	0.023	2.909365	15.63
5,7-Dihydroxyflavone	C10028	HMDB0036619	NA	NA	D-CKD	1.87	0.031	2.025846	21.32
L-Proline	C00148	HMDB0000162	Yes	Yes	D-CKD	2.02	0.025	2.009057	22.51
Hexamethylquercetagetin	NA	HMDB0029308	NA	NA	D-CKD	1.88	0.041	0.422767	27.33
5,7,3′-Trihydroxy-4′-methoxyflavanone	NA	HMDB0030746	NA	NA	D-CKD	1.74	0.038	0.411529	24.55
1,3,7-Trimethyluric acid	C16361	HMDB0002123	Yes	Yes	D-CKD	2.15	0.031	0.363065	18.55
Homocarnosine	C00884	HMDB0000745	Yes	Yes	D-CKD	2.41	0.029	0.173692	17.21
Epinephrine	C00788	HMDB0000068	Yes	Yes	D-CKD	2.37	0.034	0.171792	16.03
Acetaminophen	C06804	HMDB0001859	NA	Yes	D-CKD	2.16	0.022	0.155547	18.45
N-Acetylputrescine	C02714	HMDB0002064	Yes	Yes	D-CKD	2.53	0.018	0.108809	13.57
Linoleic acid	C01595	HMDB0000673	Yes	Yes	D-CKD	2.77	0.021	0.089211	14.81
Avocadyne	NA	HMDB0035473	NA	NA	D-CKD	2.63	0.019	0.063397	9.88
Ephedrine	C01575	HMDB0015451	Yes	Yes	D-CKD	3.04	0.015	0.052377	10.71
Octadecylamine	NA	HMDB0029586	NA	NA	D-CKD	2.87	0.009	0.026035	8.54

**FIG 7:**
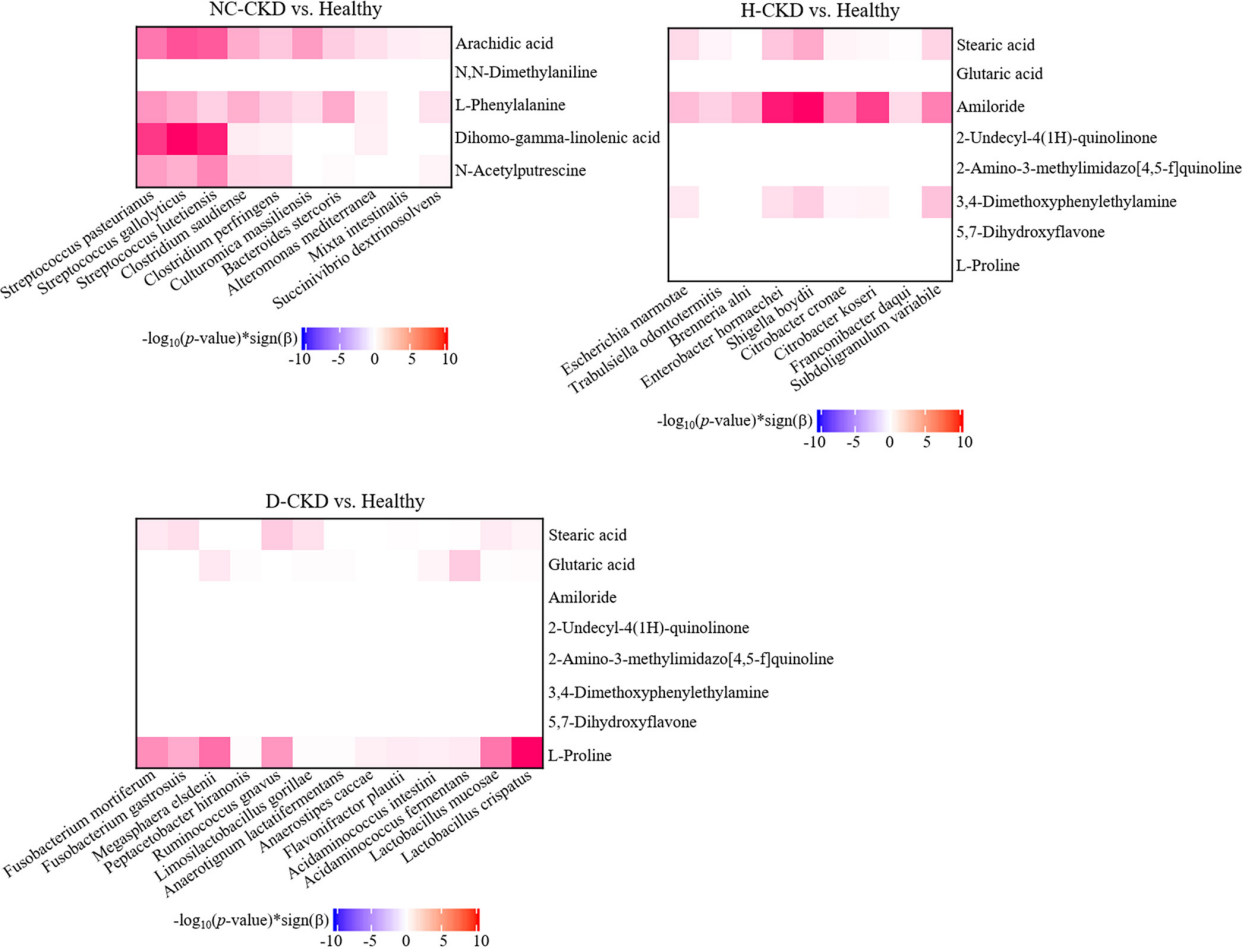
Associations among CKD-enriched gut metabolites and identified OTUs in enrolled CKD patients. The association between identified metabolites and OTUs along with CKD occurrence is shown in a Heatmap. The metabolites-OTUs associations were evaluated with the utilization of zero-inflated negative binomial (ZINB) regressions. The strengths of associations were measured by -log_10_(*P*-value)*sign(Beta) from the results of ZINB regressions and *P* value < 0.05 was identified as the cut-off for significance.

To evaluate the impact of altered gut microbiota or metabolite on pathogenesis of CKD, the association between altered metabolite profile and microbial community identified in each CKD group was demonstrated with the utilization of the Zero-inflated negative binomial (ZINB) regression (R package pscl) (24). Among the identified OTUs or metabolite discriminating in distinct CKD group from healthy participants, the significant associations between Streptococcus, *Clostridium*, *Culturomica*, and *Bacteroides* genera and 4 NC-CKD-enriched metabolites, including Arachidic acid, L-Phenylalanine, Dihomo-gamma-linolenic acid, and N-Acetylputrescine were identified with NC-CKD occurrence (Fig. 7, left, *P < *0.05). The convincing correlations of *Fusobacterium* genera, Megasphaera elsdenii, Ruminococcus gnavus, and *Lactobacillus* genera with l-Proline and Stearic acid were identified to discriminate the d-CKD patients from the healthy participants (Fig. 7, right, *P < *0.05). In H-CKD groups, the close associations of relatively abundant Stearic acid, Amiloride, and 3,4-Dimethoxyphenylethylamine with the identified OTUs, including Escherichia marmotae, Enterobacter hormaechei, Shigella boydii, Citrobacter koseri, and Subdoligranulum variabile were identified (Fig. 7, middle, *P < *0.05). These results suggested the potential relevance between species-OTU associations and the diagnosis of CKD with distinct pathogenic factor.

### Predictive utility of species-metabolite association to the causation between CKD and risk factor.

**FIG 8:**
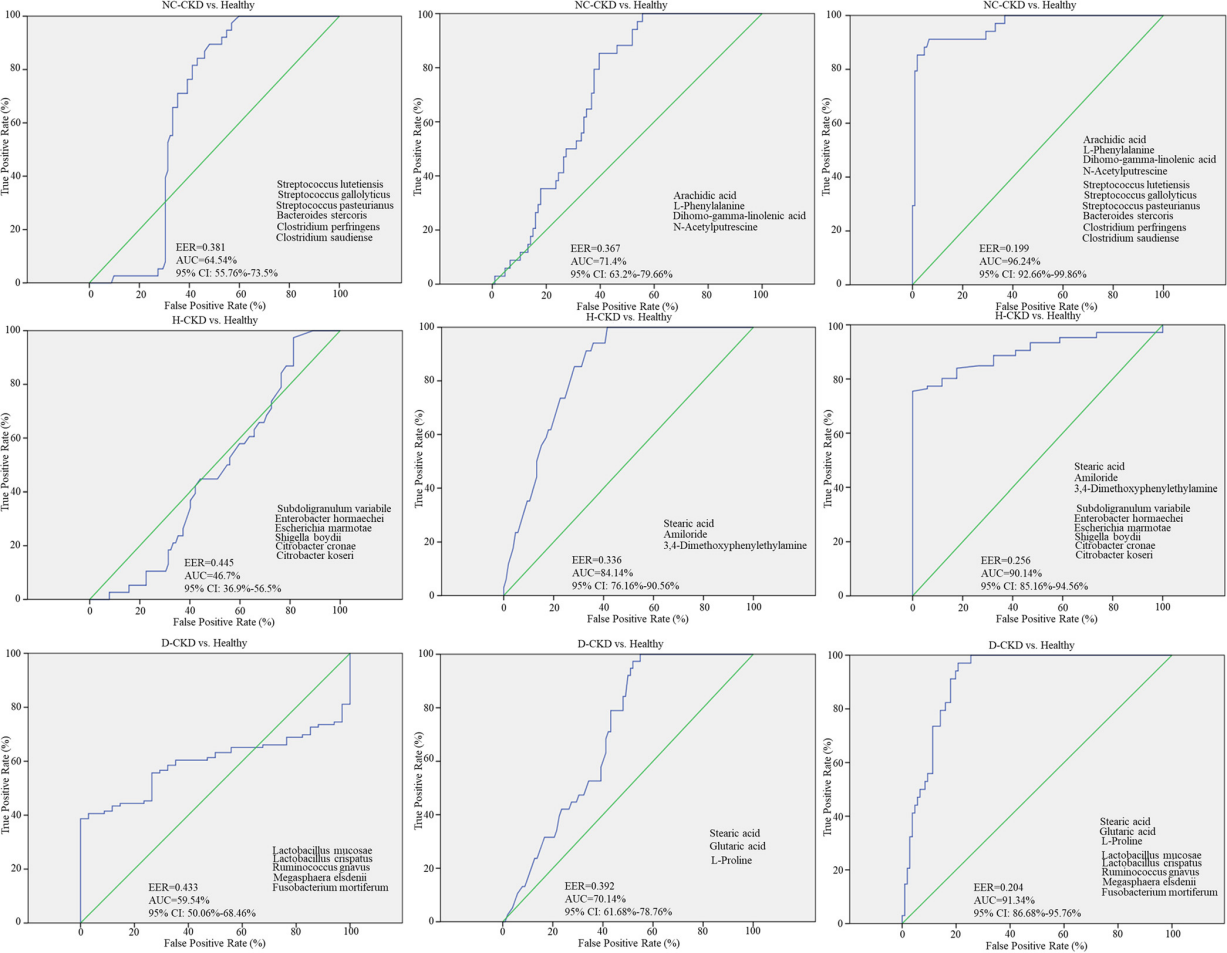
Predictive performance of gut microbiota or metabolite toward the occurrence of CKD with distinct pathogenic factor was evaluated using the random forests model. The area under the receiver operating characteristics (ROC) curve (AUC) was utilized to distinguish the enrolled diabetic CKD (d-CKD), hypertensive CKD (H-CKD), or CKD patients with no comorbidity (NC-CKD), from the healthy participants with the discriminative abundances of identified OTUs (left), the changes in intensity of gut metabolites (middle), or strength of OTUs-metabolites association (right).

The utility of characterized species-metabolite association on distinguishing CKD patients (*n *=* *96) from healthy counterparts (*n *=* *60) that recruited in our previous study was subsequently evaluated by using a random forest regression model (25). The results of receiver operating characteristics (ROC) curve were generated with the relative abundance of identified OTUs, CKD-enriched metabolites, or the strength of OTU-metabolite association in individual CKD group. The relevant OTUs discriminated NC-CKD (*n *=* *40), H-CKD (*n *=* *26), or d-CKD patients (*n *=* *30) from healthy group with an area under the ROC curve (AUC) from 0.645, 0.467, or 0.595 (Fig. 8, left). The utility of CKD-enriched metabolites at distinguishing distinct CKD groups from healthy participants were identified with an AUC from 0.714, 0.841, or 0.701 (Fig. 8, middle). The specific OTU-metabolite associations were shown to achieve convincing discrimination between healthy participants and CKD patients with ROC analyses. Utilization of the specific species-metabolite association discriminated NC-CKD, H-CKD, or d-CKD patients from healthy condition with an AUC value from 0.962, 0.901, or 0.913 (Fig. 8, right).

## DISCUSSION

A growing body of studies suggest the implication of gut dysbiosis and altered metabolite composition in the pathogenesis of CKD with underlying phenomenon, including mucosal permeability, systematic immunity, or metabolic disorders. With advancements in dual-omics approaches, identification of gut microbiota or metabolite profile allows the analysis of causation resulting in the deterioration of CKD. In this study, the specific gut dysbiosis, altered metabolite profile, and its association were characterized in the sub-population of CKD patients.

Hypertension is considered a well-known and critical risk factor toward CKD occurrence, and the related cohort study documented the association between hypertension and approximately 90% of patients diagnosed with stage 3 to stage 5 CKD (26). Gut dysbiosis was identified in hypertensive animal model or patients diagnosed with hypertension (27, 28). In brief, increases in the opportunistic pathogens, including *Enterococcus*, *Enterobacteriaceae*, and Klebsiella genera were identified under the hypertensive condition (29, 30). In contrast, the medical intervention toward gut dysbiosis, such as supplementation with high-fiber diet or probiotics, was demonstrated to exhibit blood pressure-lowering effect in the animal model or clinical patients (31, 32). In addition to hypertension, gut dysbiosis was characterized as well in patients diagnosed with diabetic kidney disease (DKD) which is a critical cause of ESRD (33). Recent studies disclosed the relevance of gut dysbiosis in richness or diversity with the occurrence of DKD (34, 35). Previous studies demonstrated a reduction in the butyrate-producing species, such as *Roseburia intestinals* or Faecalibacterium prausnitzii, with a concomitant increase in the opportunistic pathogen, including *Bacteroides*, *Clostridium*, *Eggerthella*, and Escherichia genera in the T2DM patients (36). The close relevance between decline in abundance of the butyrate-producers and glycemic condition with anti-diabetic intervention was documented, which suggest the application of microbial signature on evaluating the severity of DM or DKD (37). The relatively high abundances of Oxalobacter Formigenes and Veillonella Parvula were relevant to the bile acid metabolism and identified in gut microbiota of H-CKD patients compared to those of healthy counterparts, which potentially exhibited predictive utility at the occurrence or early stage of H-CKD (38). Similarly, the relatively abundant levels of four lipopolysaccharide producing Gram-negative phyla, including *Bacteroidetes*, *Proteobacteria*, *Verrucomicrobia* and *Fusobacteria* were determined in the gut microbiota of the T2DM-CKD compared with healthy groups (39). Nevertheless, the species-level resolution toward identification of d-CKD-related microorganisms and predictive utility of these candidate toward the occurrence of d-CKD should be further determined.

Trimethylamine N-oxide (TMAO) is an amine compound generated by gut microbial community with the carnitine-rich food and subsequently oxidized by liver-produced flavin monooxygenase 3 (40). The correlation between high serum level of TMAO and hypertension has been widely proven a major and critical causative factor involved in hypertensive CKD (41). In our study, the relatively high abundance of carnitine and TMAO was commonly characterized among all CKD patients, which suggested the impact of high TMAO on occurrence of CKD with distinct comorbidity. In contrast, the beneficial short chain fatty acid (SCFA) is produced by gut bacteria with the metabolism of fiber-rich food, which exerted protective actions against inflammation, metabolic disorders, or dysfunction in mice or rat models (42, 43). Analytic results of microbial communities suggested that supplementation of high-fiber diets manipulated the DM-associated dysbiosis with the reduced growth of the pathobiont, such as urease-produced Bilophila wadsworthia, and elevated abundances of SCFA-producing probiotics, including *Prevotella* and *Bifidobacterium* in the streptozotocin-induced diabetes model (44). Among the derived SCFAs, butyrate has been widely reported to exhibit defensive impact against oxidative stress, strengthen mucosal permeability, or hamper nephropathy progression through epigenetic regulation, subsequently accounting for renal dysfunction in DKD (45, 46). In this study, the increases in relative abundances of bile acid and saturated fatty acid, such as allocholic acid or stearic acid, were characterized among all CKD patients or specifically identified in d-CKD patients, which suggested the close relevance between dietary intake and severity or progression of d-CKD.

Alteration in gut microbiota has been considered an important factor in manipulating the production of toxins involved in CKD-associated complications (47), which may function as the disease-specific biomarker as well. In our study, the discriminative gut microbiota-metabolite association was demonstrated to exert the most specific utility to differentiate CKD patient from healthy group. In addition, the influence of altered metabolite profile on renal function through particular pathway suggested its application for mechanistic investigation or clinical intervention. The gut microbiota or metabolite profile is diverse in different region or race with the dietary intake, life style, or study pipeline. Therefore, this distinction is critical and worthy of further pursue for the development of personal medicine toward CKD patient.

### Conclusion.

In this study, utilization of dual-omics approach provides high-resolution gut dysbiosis and altered metabolite profiles which could function as a comorbidity-associated marker for the early prevention, screening, or design of medical intervention to the CKD patient or high-risk population. Prior to the potential achievement, a longitudinal and subsequent functional study is essential for better understanding of the causative effect of altered gut microbiota or metabolite on the pathogenesis of CKD associated with distinct risk factor.

## MATERIALS AND METHODS

### Ethics statement of sample collection.

Enrollment of clinical participants, fecal sample collection, and the dual-omics assays were conducted according to the guidelines of the Declaration of Helsinki, which has been approved by the Institutional Review Board of Taipei Medical University (approval no. N202003133). Patients diagnosed with CKD were recruited from the Division of Nephrology at Taipei Municipal WanFang Hospital, and healthy participants were enrolled from the Health Examination Center at Taipei Municipal WanFang Hospital. CKD was defined in accordance with 2002 clinical practice guidelines (48). Comprehensive physical and biochemistry examination were conducted on all participants to ensure related condition with respect to kidney function, diabetes, and hypertension. A standard questionnaire was applied to evaluate lifestyle of all participants.

### Extraction of total genomic DNA in feces.

Feces sample was *in vitro* collected and stored using DNA/RNA Shield Fecal Collection tubes (Zymo Research, Irvine, CA, USA) to diminish environmental disturbance. Total genomic DNA was prepared using a Quick-DNA Fecal/Soil Microbe Microprep Kit (Zymo Research, Irvine, CA, USA) according to the manufacturer’s instructions. Prior to functional analysis, the extracted DNA sample was quantified using a fluorometric assay (GeneCopoeia, Rockville, MD, USA).

### 16S ribosomal (r)RNA gene sequencing.

Gut microbiota was identified using a long-read sequencing platform. In brief, 10 ng total gDNA was subjected for library construction of 16S ribosomal (r)RNA gene using the SQK-16S024 Barcoding kit (Oxford Nanopore Technologies (ONT), Oxford, UK) according to the manufacturer’s protocol. The barcoded *16S rRNA* gene was washed and eluted from the magnetic beads (AMPure XP, Beckman Coulter, High Wycombe, UK). Pooled library containing 2 ng barcoded DNA of each participant was ligated with the adapter and sequenced on MinION flow cells (FLO-MIN106D R9.4.1; MinION instrument; ONT). The average read number of each sample was 100,000 to meet an even and sufficient reading depth toward identification of gut microbial community.

### Processing, annotation, and statistical analysis of sequencing results.

The quality check and clustering of OTU from raw reads was performed using CLC genomics workbench (Qiagen v22.0.2; CLC bio, Aarhus, Denmark) and Microbial Genomics Module v22.1 (Qiagen). Low depth samples (< 10,000 reads per sample) and low read number (< 4,000 reads per output file) were excluded from the analysis. Default parameters were used for identification and removal of chimeric reads using CLC genomics workbench. Qualified reads were mapped to 20,959 complete *16S rRNA* gene reference sequence curated from the Bacterial 16S rRNA RefSeq Targeted Loci Project (Accession No. PRJNA33175, NCBI) with 97% similarity by using Minimap2 program. The OUT table was subjected to the construction of phylogenetic tree using MUSCLE 2.0 and Maximum Likelihood Phylogeny tools available at CLC Genomics Workbench. Alpha diversity metrics (Simpson and Shannon indices) was estimated by using the phyloseq R package based on rarefied OTU table. The weighted UniFrac or Bray-Curtis indices was defined with the pipeline to indicate the inter-sample dissimilarity (beta diversity). Differential abundance of identified taxa between each group was synchronously assessed using the linear discriminant analysis (LDA) effect size (LEfSe) method with default settings on the website algorithm (https://huttenhower.sph.harvard.edu/galaxy/root). The relative difference of identified taxa was identified significantly discriminative with a *P* value < 0.05 and an LDA score (log_10_) > 3 or <-3.

### Extraction of fecal metabolites.

The extraction of fecal metabolites was commissioned to a commercial company (BIOTOOLS Co., Ltd.; Taipei, Taiwan). In brief, 50 mg feces sample was mixed in 1 mL extract solution (acetonitrile: methanol: water = 2: 2: 1) with vigorous vortex. The mixture was homogenized, sonicated, and incubated at −20°C for 1 h. The supernatant was separated with centrifugation (12000 rpm for 15 min at 4°C) and then transferred to a glass vial.

### Untargeted metabolomics analysis.

In this study, the untargeted identification of fecal metabolites was commissioned to a commercial company (BIOTOOLS Co., Ltd.; Taipei, Taiwan). In brief, 10 μL of extract prepared from each sample was applied to a vanquish focused ultra-performance liquid chromatography (UPLC) coupled with an Orbitrap Elite Mass Spectrometry (Thermo Fisher Scientific; San Jose, CA, USA). The binary mobile phase was composed of deionized water containing 0.1% formic acid (solvent A) and LC-MS grade acetonitrile with 0.1% formic acid (solvent B). Throughout the linear gradient elution, the percentage of Solvent B was linearly increased from 5% to 100% for 7 min, kept constant for 3 min, then decreased to 5% in 1 min. Blank injection was applied to diminish carry-over and QC application was conducted to normalize the peak area. Mass spectrometry data were harvested in positive mode with a default data-dependent acquisition set. One MS full scan was applied in a profile mode at 60000 resolution, followed by 10 data-dependent MS2 scans at 15000 resolution. The mass scan range was set from 70 to 1000 *m/z* and the normalized collision energy (NCE) was set to 25. The spray voltage, and the capillary temperature, the sheath gas and the aux gas was applied with the default set.

### Processing, annotation, and statistical analysis of UPLC-MS/MS data.

The raw data was converted to the mzXML format using ProteoWizard software for following analysis. The converted results were processed with an in-house program based on XCMS using R program for peak detection, extraction, alignment, and integration (BIOTOOLS Co., Ltd.; Taipei, Taiwan). An in-house MS2 data-base (BiotreeDB; BIOTOOLS Co., Ltd.; Taipei, Taiwan) was applied for the annotation of metabolomic profile, of which the cut-off was set to 0.3.

### Statistical analysis.

Statistic results to the generated data were shown as the mean ± standard error (SEM). Continuous variables of this study is evaluated using a one-way analysis of variance (ANOVA) coupled with Tukey’s multiple comparison *post hoc* test. A variable was identified significant with a *P* value of <0.05 (*, *P < *0.05; **, *P < *0.01; ***, *P < *0.005). The species-metabolite association under specific condition was assessed using zero-inflated negative binomial (ZINB) regression (R package pscl). The read number of identified species was defined as a dependent variable and the strength of identified metabolite was defined as an independent variable in ZINB regressions. The association was shown by -log_10_(*P*-value)*sign (β), of which β presented the regression of the metabolite. The utility of species, metabolite, or its association for predicting the diagnosis of CKD was evaluated with the results of the receiver operating characteristic (ROC) curve and area under the ROC curve (AUC) ratio generated by using SPSS Statistics 19 (IBM, Armonk, NY). The relevance between OTU number and equal error rate (EER) was evaluated by using the built-in RFCV function of the randomForest package (R version. 4.6–14). A cross-validation step was subjected to minimize the EER with an optimal predictor number in this study (49).

### Data availability.

Raw *16S rRNA* sequencing data sets were deposited with the NCBI BioProject database under accession number PRJNA899930.

## Supplementary Material

Reviewer comments

## References

[1] Luyckx VA, Tonelli M, Stanifer JW. 2018. The global burden of kidney disease and the sustainable development goals. Bull World Health Organ 96:414–422D. doi:10.2471/BLT.17.206441. 29904224 PMC5996218

[2] Kazancioglu R. 2011. Risk factors for chronic kidney disease: an update. Kidney Int Suppl 2013:368–371. 10.1038/kisup.2013.79 PMC4089662 25019021

[3] Amatruda M, Gembillo G, Giuffrida AE, Santoro D, Conti G. 2021. The aggressive diabetic kidney disease in youth-onset type 2 diabetes: pathogenetic mechanisms and potential therapies. Medicina 57:868. doi:10.3390/medicina57090868. 34577791 PMC8467670

[4] Giandalia A, Giuffrida AE, Gembillo G, Cucinotta D, Squadrito G, Santoro D, Russo GT. 2021. Gender differences in diabetic kidney disease: focus on hormonal, genetic and clinical factors. Int J Mol Sci 22:5808. doi:10.3390/ijms22115808. 34071671 PMC8198374

[5] Alicic RZ, Rooney MT, Tuttle KR. 2017. Diabetic kidney disease: challenges, progress, and possibilities. Clin J Am Soc Nephrol 12:2032–2045. doi:10.2215/CJN.11491116. 28522654 PMC5718284

[6] Mafra D, Borges NA, Lindholm B, Shiels PG, Evenepoel P, Stenvinkel P. 2021. Food as Medicine: Targeting the Uraemic Phenotype in Chronic Kidney Disease. Nat Rev Nephrol 17:153–171. doi:10.1038/s41581-020-00345-8. 32963366

[7] Dinan TG, Cryan JF. 2017. Gut-brain axis in 2016: Brain-gut-microbiota axis — mood, metabolism and behaviour. Nat Rev Gastroenterol Hepatol 14:69–70. doi:10.1038/nrgastro.2016.200. 28053341

[8] Evenepoel P, Poesen R, Meijers B. 2017. The gut-kidney axis. Pediatr Nephrol 32:2005–2014. doi:10.1007/s00467-016-3527-x. 27848096

[9] Rooks MG, Garrett WS. 2016. Gut microbiota, metabolites and host immunity. Nat Rev Immunol 16:341–352. doi:10.1038/nri.2016.42. 27231050 PMC5541232

[10] Bravo JA, Forsythe P, Chew MV, Escaravage E, Savignac HM, Dinan TG, Bienenstock J, Cryan JF. 2011. Ingestion of Lactobacillus strain regulates emotional behavior and central GABA receptor expression in a mouse via the vagus nerve. Proc Natl Acad Sci USA 108:16050–16055. doi:10.1073/pnas.1102999108. 21876150 PMC3179073

[11] Poesen R, Claes K, Evenepoel P, de Loor H, Augustijns P, Kuypers D, Meijers B. 2016. Microbiota-Derived Phenylacetylglutamine Associates with Overall Mortality and Cardiovascular Disease in Patients with CKD. J Am Soc Nephrol 27:3479–3487. doi:10.1681/ASN.2015121302. 27230658 PMC5084895

[12] Aron-Wisnewsky J, Clément K. 2016. The Gut Microbiome, Diet, and Links to Cardiometabolic and Chronic Disorders. Nat Rev Nephrol 12:169–181. doi:10.1038/nrneph.2015.191. 26616538

[13] Caggiano G, Cosola C, Di Leo V, Gesualdo M, Gesualdo L. 2020. Microbiome Modulation to Correct Uremic Toxins and to Preserve Kidney Functions. Curr Opin Nephrol Hypertens 29:49–56. doi:10.1097/MNH.0000000000000565. 31725010

[14] Nallu A, Sharma S, Ramezani A, Muralidharan J, Raj D. 2017. Gut Microbiome in Chronic Kidney Disease: Challenges and Opportunities. Transl Res 179:24–37. doi:10.1016/j.trsl.2016.04.007. 27187743 PMC5086447

[15] Yoshifuji A, Wakino S, Irie J, Tajima T, Hasegawa K, Kanda T, Tokuyama H, Hayashi K, Itoh H. 2016. Gut Lactobacillus Protects against the Progression of Renal Damage by Modulating the Gut Environment in Rats. Nephrol Dial Transplant 31:401–412. doi:10.1093/ndt/gfv353. 26487672

[16] Yang CY, Chen TW, Lu WL, Liang SS, Huang HD, Tseng CP, Tarng DC. 2021. Synbiotics alleviate the gut indole load and dysbiosis in chronic kidney disease. Cells 10:114. 33435396 10.3390/cells10010114 PMC7826693

[17] Tang WH, Wang Z, Kennedy DJ, Wu Y, Buffa JA, Agatisa-Boyle B, Li XS, Levison BS, Hazen SL. 2015. Gut Microbiota-Dependent Trimethylamine N-Oxide (TMAO) Pathway Contributes to Both Development of Renal Insufficiency and Mortality Risk in Chronic Kidney Disease. Circ Res 116:448–455. doi:10.1161/CIRCRESAHA.116.305360. 25599331 PMC4312512

[18] Huang Y, Zhou J, Wang S, Xiong J, Chen Y, Liu Y, Xiao T, Li Y, He T, Li Y, Bi X, Yang K, Han W, Qiao Y, Yu Y, Zhao J. 2020. Indoxyl Sulfate Induces Intestinal Barrier Injury Through IRF1-DRP1 Axis-Mediated Mitophagy Impairment. Theranostics 10:7384–7400. doi:10.7150/thno.45455. 32641998 PMC7330852

[19] Huang H, Li K, Lee Y, Chen M. 2021. Preventive Effects of Lactobacillus Mixture against Chronic Kidney Disease Progression through Enhancement of Beneficial Bacteria and Downregulation of Gut-Derived Uremic Toxins. J Agric Food Chem 69:7353–7366. doi:10.1021/acs.jafc.1c01547. 34170659

[20] Iwashita Y, Ohya M, Yashiro M, Sonou T, Kawakami K, Nakashima Y, Yano T, Iwashita Y, Mima T, Negi S, Kubo K, Tomoda K, Odamaki T, Shigematsu T. 2018. Dietary Changes Involving Bifidobacterium longum and Other Nutrients Delays Chronic Kidney Disease Progression. Am J Nephrol 47:325–332. doi:10.1159/000488947. 29779028

[21] Shin J, Lee S, Go MJ, Lee SY, Kim SC, Lee CH, Cho BK. 2016. Analysis of the mouse gut microbiome using full-length 16S rRNA amplicon sequencing. Sci Rep 6:29681. doi:10.1038/srep29681. 27411898 PMC4944186

[22] Somerville V, Lutz S, Schmid M, Frei D, Moser A, Irmler S, Frey JE, Ahrens CH. 2019. Long-read based de novo assembly of low-complexity metagenome samples results in finished genomes and reveals insights into strain diversity and an active phage system. BMC Microbiol 19:143. doi:10.1186/s12866-019-1500-0. 31238873 PMC6593500

[23] Su J, Li CX, Liu HY, Lian QY, Chen A, You ZX, Li K, Cai YH, Lin YX, Pan JB, Zhang GX, Ju CR, You CX, He JX. 2022. The Airway Microbiota Signatures of Infection and Rejection in Lung Transplant Recipients. Microbiol Spectr 10:e0034421. doi:10.1128/spectrum.00344-21. 35416686 PMC9045364

[24] Fang R, Wagner BD, Harris JK, Fillon SA. 2016. Zero-inflated negative binomial mixed model: an application to two microbial organisms important in oesophagitis. Epidemiol Infect 144:2447–2455. doi:10.1017/S0950268816000662. 27049299 PMC9150531

[25] Chen TH, Liu CW, Ho YH, Huang CK, Hung CS, Smith BH, Lin JC. 2021. Gut Microbiota Composition and Its Metabolites in Different Stages of Chronic Kidney Disease. JCM 10:3881. doi:10.3390/jcm10173881. 34501329 PMC8432073

[26] Rao MV, Qiu Y, Wang C, Bakris G. 2008. Hypertension and CKD: Kidney Early Evaluation Program (KEEP) and National Health and Nutrition Examination Survey (NHANES), 1999–2004. Am J Kidney Dis 51:S30–S37. doi:10.1053/j.ajkd.2007.12.012. 18359406

[27] Yang T, Santisteban MM, Rodriguez V, Li E, Ahmari N, Carvajal JM, Zadeh M, Gong M, Qi Y, Zubcevic J, Sahay B, Pepine CJ, Raizada MK, Mohamadzadeh M. 2015. Gut dysbiosis is linked to hypertension. Hypertension 65:1331–1340. doi:10.1161/HYPERTENSIONAHA.115.05315. 25870193 PMC4433416

[28] Li J, Zhao F, Wang Y, Chen J, Tao J, Tian G, Wu S, Liu W, Cui Q, Geng B, Zhang W, Weldon R, Auguste K, Yang L, Liu X, Chen L, Yang X, Zhu B, Cai J. 2017. Gut microbiota dysbiosis contributes to the development of hypertension. Microbiome 5:14. doi:10.1186/s40168-016-0222-x. 28143587 PMC5286796

[29] Jiang S, Xie S, Lv D, Wang P, He H, Zhang T, Zhou Y, Lin Q, Zhou H, Jiang J, Nie J, Hou F, Chen Y. 2017. Alteration of the gut microbiota in Chinese population with chronic kidney disease. Sci Rep 7:2870. doi:10.1038/s41598-017-02989-2. 28588309 PMC5460291

[30] Wang F, Jiang H, Shi K, Ren Y, Zhang P, Cheng S. 2012. Gut bacterial translocation is associated with microinflammation in end-stage renal disease patients. Nephrology (Carlton) 17:733–738. doi:10.1111/j.1440-1797.2012.01647.x. 22817644

[31] Marques FZ, Nelson E, Chu PY, Horlock D, Fiedler A, Ziemann M, Tan JK, Kuruppu S, Rajapakse NW, El-Osta A, Mackay CR, Kaye DM. 2017. High-fiber diet and acetate supplementation change the gut microbiota and prevent the development of hypertension and heart failure in hypertensive mice. Circulation 135:964–977. doi:10.1161/CIRCULATIONAHA.116.024545. 27927713

[32] Khalesi S, Sun J, Buys N, Jayasinghe R. 2014. Effect of probiotics on blood pressure: a systematic review and meta-analysis of randomized, controlled trials. Hypertension 64:897–903. doi:10.1161/HYPERTENSIONAHA.114.03469. 25047574

[33] Gheith O, Farouk N, Nampoory N, Halim MA, Al-Otaibi T. 2016. Diabetic kidney disease: world wide difference of prevalence and risk factors. J Nephropharmacol 5:49–56. 28197499 PMC5297507

[34] Scheithauer TPM, Rampanelli E, Nieuwdorp M, Vallance BA, Verchere CB, van Raalte DH, Herrema H. 2020. Gut microbiota as a trigger for metabolic inflammation in obesity and type 2 diabetes. Front Immunol 11:571731. doi:10.3389/fimmu.2020.571731. 33178196 PMC7596417

[35] Le Chatelier E, Nielsen T, Qin J, Prifti E, Hildebrand F, Falony G, Almeida M, Arumugam M, Batto JM, Kennedy S, Leonard P, Li J, Burgdorf K, Grarup N, Jørgensen T, Brandslund I, Nielsen HB, Juncker AS, Bertalan M, Levenez F, Pons N, Rasmussen S, Sunagawa S, Tap J, Tims S, Zoetendal EG, Brunak S, Clément K, Doré J, Kleerebezem M, Kristiansen K, Renault P, Sicheritz-Ponten T, de Vos WM, Zucker JD, Raes J, Hansen T, Bork P, Wang J, Ehrlich SD, Pedersen O, MetaHIT consortium. 2013. Richness of human gut microbiome correlates with metabolic markers. Nature 500:541–546. doi:10.1038/nature12506. 23985870

[36] Karlsson FH, Tremaroli V, Nookaew I, Bergström G, Behre CJ, Fagerberg B, Nielsen J, Bäckhed F. 2013. Gut metagenome in European women with normal, impaired and diabetic glucose control. Nature 498:99–103. doi:10.1038/nature12198. 23719380

[37] Wu H, Tremaroli V, Schmidt C, Lundqvist A, Olsson LM, Krämer M, Gummesson A, Perkins R, Bergström G, Bäckhed F. 2020. The gut microbiota in prediabetes and diabetes: a population-based cross-sectional study. Cell Metab 32:379–390.e3. doi:10.1016/j.cmet.2020.06.011. 32652044

[38] Li X, Wang L, Ma S, Lin S, Wang C, Wang H. 2022. Combination of oxalobacter formigenes and veillonella parvula in gastrointestinal microbiota related to bile-acid metabolism as a biomarker for hypertensive nephropathy. Int J Hypertens 2022:5999530. 35620320 10.1155/2022/5999530 PMC9129936

[39] Salguero MV, Al-Obaide MAI, Singh R, Siepmann T, Vasylyeva TL. 2019. Dysbiosis of Gram-negative gut microbiota and the associated serum lipopolysaccharide exacerbates inflammation in type 2 diabetic patients with chronic kidney disease. Exp Ther Med 18:3461–3469. doi:10.3892/etm.2019.7943. 31602221 PMC6777309

[40] Livshits G, Kalinkovich A. 2019. Inflammaging as a common ground for the development and maintenance of sarcopenia, obesity, cardiomyopathy and dysbiosis. Ageing Res Rev 56:100980. doi:10.1016/j.arr.2019.100980. 31726228

[41] Zhang L, Xie F, Tang H, Zhang X, Hu J, Zhong X, Gong N, Lai Y, Zhou M, Tian J, Zhou Z, Xie L, Hu Z, Zhu F, Jiang J, Nie J. 2022. Gut microbial metabolite TMAO increases peritoneal inflammation and peritonitis risk in peritoneal dialysis patients. Transl Res 240:50–63. doi:10.1016/j.trsl.2021.10.001. 34673277

[42] Morrison DJ, Preston T. 2016. Formation of short chain fatty acids by the gut microbiota and their impact on human metabolism. Gut Microbes 7:189–200. doi:10.1080/19490976.2015.1134082. 26963409 PMC4939913

[43] Andrade-Oliveira V, Amano MT, Correa-Costa M, Castoldi A, Felizardo RJ, de Almeida DC, Bassi EJ, Moraes-Vieira PM, Hiyane MI, Rodas AC, Peron JP, Aguiar CF, Reis MA, Ribeiro WR, Valduga CJ, Curi R, Vinolo MA, Ferreira CM, Câmara NO. 2015. Gut bacteria products prevent AKI induced by ischemia-reperfusion. J Am Soc Nephrol 26:1877–1888. doi:10.1681/ASN.2014030288. 25589612 PMC4520159

[44] Li YJ, Chen X, Kwan TK, Loh YW, Singer J, Liu Y, Ma J, Tan J, Macia L, Mackay CR, Chadban SJ, Wu H. 2020. Dietary fiber protects against diabetic nephropathy through short-chain fatty acid-mediated activation of G protein-coupled receptors GPR43 and GPR109A. J Am Soc Nephrol 31:1267–1281. doi:10.1681/ASN.2019101029. 32358041 PMC7269358

[45] Ruiz S, Pergola PE, Zager RA, Vaziri ND. 2013. Targeting the transcription factor Nrf2 to ameliorate oxidative stress and inflammation in chronic kidney disease. Kidney Int 83:1029–1041. doi:10.1038/ki.2012.439. 23325084 PMC3633725

[46] Felizardo RJF, de Almeida DC, Pereira RL, Watanabe IKM, Doimo NTS, Ribeiro WR, Cenedeze MA, Hiyane MI, Amano MT, Braga TT, Ferreira CM, Parmigiani RB, Andrade-Oliveira V, Volpini RA, Vinolo MAR, Mariño E, Robert R, Mackay CR, Camara NOS. 2019. Gut microbial metabolite butyrate protects against proteinuric kidney disease through epigenetic- and GPR109a-mediated mechanisms. FASEB J 33:11894–11908. doi:10.1096/fj.201901080R. 31366236

[47] Wikoff WR, Anfora AT, Liu J, Schultz PG, Lesley SA, Peters EC, Siuzdak G. 2009. Metabolomics analysis reveals large effects of gut microflora on mammalian blood metabolites. Proc Natl Acad Sci USA 106:3698–3703. doi:10.1073/pnas.0812874106. 19234110 PMC2656143

[48] Levey AS, Eckardt KU, Tsukamoto Y, Levin A, Coresh J, Rossert J, De Zeeuw D, Hostetter TH, Lameire N, Eknoyan G. 2005. Definition and classification of chronic kidney disease: a position statement from kidney disease: improving global outcomes (KDIGO). Kidney Int 67:2089–2100. doi:10.1111/j.1523-1755.2005.00365.x. 15882252

[49] Leo Breiman L. 2001. Random Forests. Mach Learn 45:5–32. doi:10.1023/A:1010933404324.

